# Alterations of CD4+T Cell Subsets in Blood and Peritoneal Fluid in Different Stages of Endometriosis 

**DOI:** 10.22074/ijfs.2020.6127

**Published:** 2020-10-12

**Authors:** Fatemeh Pashizeh, Reza Mansouri, Fatemeh Davari-Tanha, Reyhaneh Hosseini, Zahra Asgari, Hamideh Aghaei, Farangis Najafi Arbastan, Samira Rajaei

**Affiliations:** 1 Department of Immunology, School of Medicine, Shahid Sadoughi University of Medical Sciences, Yazd, Iran; 2 Department of Immunology, School of Medicine, International Campus, Shahid Sadoughi University of Medical Sciences, Yazd, Iran; 3 Department of Obstetrics and Gynaecology, Yas Hospital, School of Medicine, Tehran University of Medical Sciences, Tehran, Iran; 4 Department of Obstetrics and Gynaecology, Research Development Centre, School of Medicine, Tehran University of Medical Sciences, Tehran, Iran; 5 Department of Obstetrics and Gynaecology, School of Medicine, Tehran University of Medical Sciences, Tehran, Iran; 6 Department of Immunology, School of Medicine, Tehran University of Medical Sciences, Tehran, Iran

**Keywords:** Endometriosis, Regulatory T Cell, T helper 1 Cell, T helper 2 Cell, T helper 17 Cell

## Abstract

**Background:**

Endometriosis is a chronic inflammatory disorder with known immune disturbances. The aim of this study was to compare the frequency of different CD4+ T cells [T helper (Th)1, Th2, Th17 and regulatory T cells (Tregs)] in peripheral blood (PB) and peritoneal fluid (PF) of patients that have early and advanced stages of endome- triosis with a control group.

**Materials and Methods:**

In this case control study, PB and PF samples were collected from women aged 24-40 years who underwent laparoscopy procedures. The frequency of CD4+ T subsets were analysed by flow cytometry and com- pared between three study groups; early endometriosis (stage I, II), advanced endometriosis (stage III, IV) and control (no endometriosis). T cell numbers were compared between the PB and PF in each of the aforementioned groups.

**Results:**

No statistically significant difference was found between the study groups regarding the numbers of Th1, Th2 and Th17 cells in PB. The PF of patients with advanced endometriosis had increased numbers of Th17 cells compared to the control group (P=0.003), with P values of 0.059 and 0.045 in both menstrual phases. Increased numbers of Th2 cells in PF from early compared to advanced stages of endometriosis were detected exclusively in the luteal phase (P=0.035). The control group had increased numbers of Treg and Th2 cells in the PF compared to PB (both, P value=0.046). However, in the early stages of endometriosis there were more Th2, Th17 and Treg cells in the PF compared to PB (P values: 0.005, 0.047 and 0.013, respectively), while the number of Th17 cells was higher in the PF compared with PB in the advanced stages of endometriosis (P= 0.013).

**Conclusion:**

There were increased numbers of Th17 cells in the PF of patients with advanced stages of endometriosis, which could be related to the severity of this disease.

## Introduction

Endometriosis, characterized by presence of endometrial tissue outside of uterine cavity, is a chronic inflammatory disorder that involves 6-10% of reproductive age women (1, 2).

Despite numerous investigations regarding the pathogenesis of endometriosis, the definite aetiology remains undetermined. Many factors such as genetic predisposition, hormonal imbalance, environmental factors and, especially, immune system disturbances are potential aetiologic factors (3-5).

Systemic and local changes in immune responses that include impaired CD4+ T cells have been frequently reported as contributing factors in endometriosis pathogenesis (6-8). CD4+ T cells exert their potential role in endometriosis through cytokines that are involved in implantation and proliferation of ectopic endometrial cells, inflammation and angiogenesis (7, 9-12). Aberrations in CD4+ T cell populations have been assessed in several endometriosis studies (11-16); however, only one or two of these CD4+ T cells were investigated in each of these studies. To date, the trend in changes in all four CD4+ T subsets in early and advanced stages of endometriosis and between peripheral blood (PB) and peritoneal fluid (PF) in each stage of this disease have not been assessed in a single study. Hence, the current study was designed and implemented to answer the following questions:

1. Are there deviations in the numbers of T helper (Th)1, Th2, Th17 and regulatory T (Treg) cells in peripheral blood between stages I, II and III, IV of endometriosis and a control group?

2. Are there deviations in the numbers of Th1, Th2, Th17 and Treg cells in PF between stages I, II and III, IV of endometriosis and a control group?

3. Are there any changes in the number of Th1, Th2, Th17 and Treg cells between blood and PF in endometriosis stages I, II and III, IV and control group?

## Materials and Methods

### Participants and specimens

This case-control study enrolled 20 women with endometriosis confirmed by observation of endometriotic lesions during laparoscopy and pathological confirmation of disease in biopsies that were taken from endometriotic foci as the case group and a control group comprised of 10 women with no evidence of endometriosis during laparoscopy. All participants were 24 to 40 years of age. The presence of endometriosis was confirmed by a gynaecologist during laparoscopy. According to the revised American Society for Reproductive Medicine classification of endometriosis (17), we divided the endometriosis group into two subgroups - 10 women with early stage endometriosis (stage I, II) and 10 women with advanced stage of this disease (stage III, IV). All control women underwent laparoscopy for other diseases (dermoid or follicular cysts) and endometriosis was not detected in any of these women. Women who had a history of autoimmunity, inflammatory disorders (including allergies) or other gynaecological diseases (e.g., polycystic ovary syndrome) and those who received hormonal treatment during three months before taking samples were excluded from the study.

The study was approved by the Ethics Committee at Tehran University of Medical Sciences (TUMS), Tehran, Iran (Ethics code: IR.TUMS.MEDICINE.REC.1395.1073). The samples were taken from women who referred to Yas and Arash hospitals, both of which are TUMS affiliated women’s hospitals. All women signed an informed consent form for study participation before entering the study. A total of 5 mL of peripheral blood (PB) was collected from the antecubital vein of each patient before they underwent general anaesthesia. PF was aspirated by the surgeon after insertion of the second trocar at the beginning of the laparoscopic procedure. The volume of PF varied from 2 to 8 mL in different cases. In each group, the samples were classified as follicular or luteal phase based on the date of the patient’s last menopausal period, which was reported by each patient at the time of sampling and confirmed by pathologic reports in cases where samples of endometriotic lesions were obtained for pathological investigations.

### Separation of mononuclear cells

PB and PF were collected in heparinized tubes and transferred in sterile, cold conditions to the laboratory where they were diluted with phosphate-buffered saline (PBS) at a 1:1 ratio. The diluted PB or PF were layered on Ficoll-Hypaque (Inno-train, Germany) and centrifuged (1000 g, 20 minutes). The cells in the interphase layer were collected and transferred into new tubes and washed completely with PBS. After discarding the supernatant, the precipitated mononuclear cells were suspended in culture medium, and the number and viability of these cells were determined by vital staining.

### Culture and staining process

PB or PF mononuclear cells were divided into two portions. One part was used for detection of Treg cells and the other for stimulation and recognition of Th1, Th2 and Th17 cells.

Treg cells were considered to be CD4+CD25+CD127- FOXP3+ cells. For determination and evaluation of the Treg cells, we stained the mononuclear cells with FITC-labelled anti-CD4, PE/Cy7-labelled anti-CD25, and APC-labelled anti-CD127 antibodies (Biolegend, CA, USA); after cell fixation and permeabilisation, the cells were stained with PE-conjugated anti-FOXP3 antibody.

For detection of Th1, Th2 and Th17 cells in PB or PF, the mononuclear cells were cultured in Roswell Park Memorial Institute medium (RPMI) 1640 (Biosera, France) supplemented with 10% FBS (Gibco, UK), 100 U/mL penicillin, and 100 μg/mL streptomycin (Gibco, UK) in 24-well cell culture plates and stimulated with phorbol myristate acetate (PMA, Sigma, St. Louise, MO, USA) at 50 ng/mL and ionomycin (Sigma-Aldrich, St. Louise, MO, USA) at 1 μg/mL concentrations and incubated at 37°C and 5% CO_2_. After an hour, 10 μg/mL Brefeldin A (eBioscience, San Diego, CA, USA) was added. The cells were harvested after a 5-hour incubation period by using PMA, Ionomycin and Brefeldin A, followed by staining with FITC-conjugated anti-CD4 antibody. After fixation and permeabilisation, intracellular cytokines were stained with PE-Cy7-conjugated anti-interferon gamma (IFNγ), APC-conjugated anti-interleukin 4 (IL-4) and PE-conjugated anti-IL-17 antibodies. CD4+IFNγ+, CD4+IL-4+ and CD4+IL-17+ cells were considered to be Th1, Th2 and Th17 cells, respectively. Isotype-matched fluoro- phore-conjugated antibodies were used as the controls.

The stained cells (10^5^ cells) were investigated by BD FACSCalibur instrument (Becton Dickinson, CA).

### Gating method

The lymphocytes were gated according to forward and side scatters, which were representative of the cell size and granularity. In the group of stimulated cells, we considered Th1 cells to be CD4+IFNγ+, Th2 were CD4+IL-4+ and Th17 were CD4+IL-17+. For Treg cell discrimination, first the CD4+CD127- cells were gated from the lymphocytes. Then, from these gated cells, CD25+FOXP3+ were specified. The percentage of the CD4+CD25+FOXP3+CD127- cells from the lymphocyte population was defined as the frequency of the Treg cells. These percentages were calculated using FlowJo software (Version 7.6.1). The gating procedure was similar for both PB and PF (Fig . 1); however, the percentage of the lymphocytes was different in PB and PF.

### Statistical analysis

Because of the non-normal distribution of the samples, we used the Kruskal-Wallis test to compare the frequency of the Th1, Th2, Th17 and Treg cells in PB and PF samples between the three groups. The Wilcoxon test was used to compare the percentage of each T cell population in each group between PB and PF. P values <0.05 were considered to be statistically significant. SPSS version 19 and GraphPad prism Version.6 software was used for data analysis and for drawing the plots.

**Fig.1:**
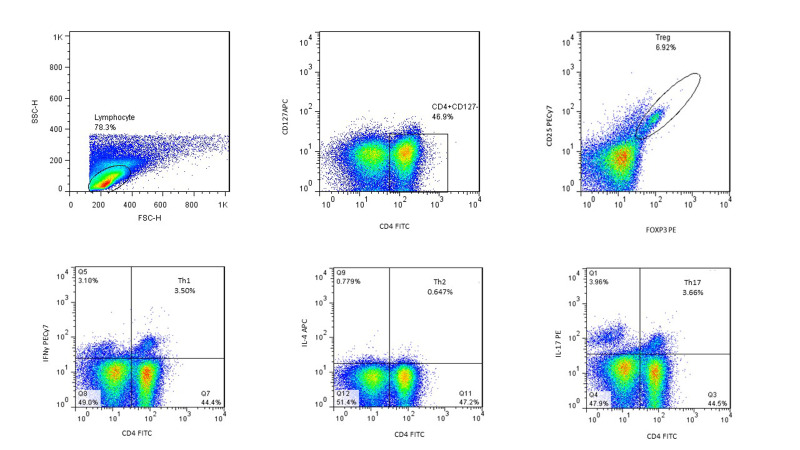
Gating strategy for the detection of regulatory T cells (Treg) (upper row) and T helper (Th)1, Th2 and Th17 cells (lower row) in peripheral blood (PB). The sample is from a patient in stage III, IV endometriosis (follicular phase). CD4+CD25+FOXP3+CD127- were defined as Treg cells. CD4+IFNγ+ were considered to be Th1 cells, CD4+IL-4+ were Th2 cells and CD4+IL-17+ were considered to be the Th17 cells.

## Results

### Participants′ characteristics

After considering the inclusion and exclusion criteria, 10 samples were selected in each group. We had six follicular and four luteal phase samples in each of the control and advanced endometriosis (stage III, IV) groups; however, in the early stages of endometriosis (stage I, II), we collected seven follicular and three luteal phase samples. The ages (median, minimum-maximum) of women in the three groups were similar: control (33.5, 27-40 years), endometriosis stage I, II (32, 24-38 years) and endometriosis stage III, IV (32, 25-38 years) (P value: 0.875). The age medians were similar between groups when compared according to menstrual phase of sampling.

### Comparison of the frequency of CD4+ T cells in blood between the three groups

Our results indicated similar numbers of Th1, Th2 and Th17 in blood samples of the three groups (P values: 0.78, 0.298, and 0.228, Fig . 2). The Kruskal-Wallis test results showed different numbers of Treg cells between the three groups (P=0.042) with mean ranks of 13.10 (control), 12.20 (stage I, II), and 21.20 (stage III, IV); however, a pairwise comparison between each pair of groups indicated that none of the P values were statistically different and the adjusted P values were 0.067 (stage I, II vs. stage III, IV of endometriosis), 0.119 (control vs. stage III, IV of endometriosis) and 1 (control vs. stage I, II of endometriosis). Our results showed no significant differences in the four blood CD4+ T cell subsets when they were compared based on menstrual phases.

### Comparison of the frequency of CD4+ T cells in peritoneal fluid between the three groups

The numbers of Th1, Th2 and Treg cells were not different in the PF of the three groups; however, there was only an increased number of Th2 cells in the PF of endometriosis stage I, II (mean rank=10) compared to stage III, IV (mean rank=3.5) in the luteal phase (P=0.035). There were increased numbers of Th17 cells in the PF of advanced endometriosis (stage III, IV) cases compared to controls (adjusted P=0.003, mean rank of 22.4 in advanced endometriosis vs. 9.30 in the control). This trend was seen in both the follicular (P=0.059) and luteal [P=0.045, mean ranks were 8.25 (advanced endometriosis) and 2.75 (control)] phases. The frequency of Th17 cells did not differ between the other groups (Figes. 2, 3).

**Fig.2:**
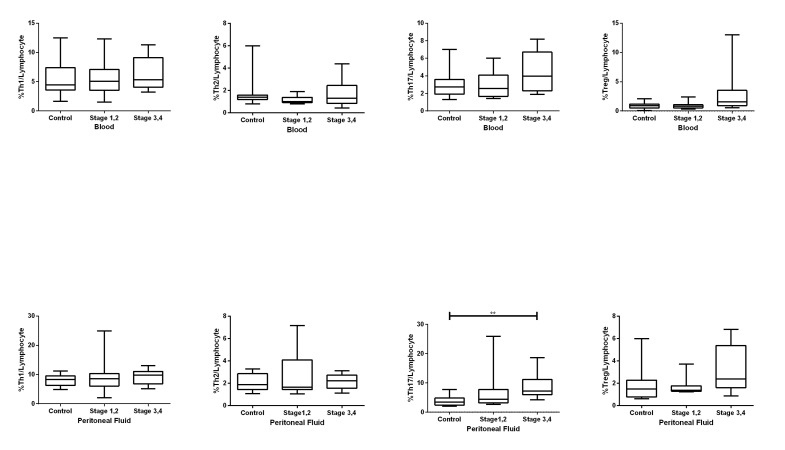
Frequency of T helper (Th)1, Th2, Th17 and regulatory T (Treg) cells compared in peripheral blood (PB) (upper row) and peritoneal fluid (PF) (lower row) amongst the control, early stages of endometriosis (stage I, II) and advanced endometriosis (stage III, IV) groups. Each box plot represents 25-75% quartiles with median.

**Fig.3:**
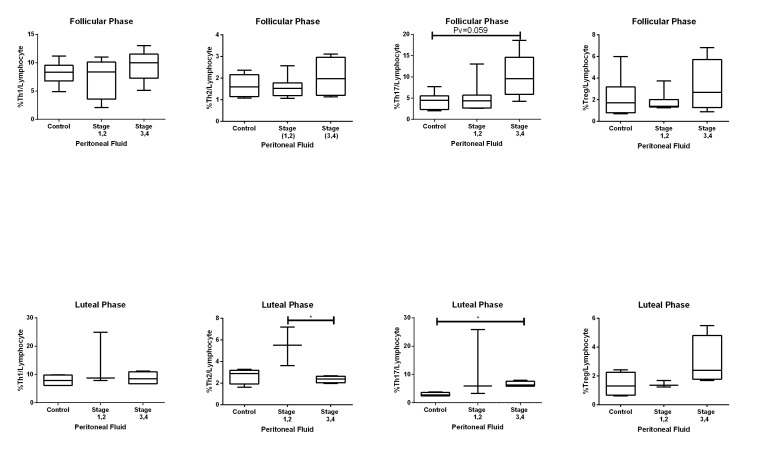
Frequency of T helper (Th)1, Th2, Th17 and regulatory T (Treg) cells in peritoneal fluid (PF) in the control, early endometriosis (stage I, II) and advanced endometriosis (stage III, IV) groups in the follicular (upper row) and luteal (lower row) phases. Each box plot represents 25-75% quartiles with the median. *; P value<0.05.

**Fig.4:**
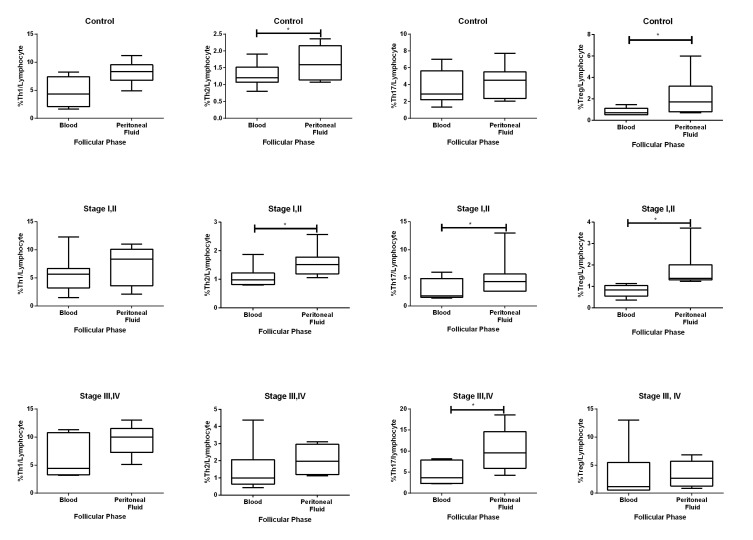
Frequency of T helper (Th)1, Th2, Th17 and regulatory T (Treg) cells were compared in peritoneal fluid (PF) with peripheral blood (PB) in the control (upper row), early endometriosis (stage I, II) (middle row) and advanced endometriosis (stage III, IV) (lower row) groups in the follicular phase. Each box plot represents 25-75% quartiles with median. *; P value<0.05.

### Comparison of the number of CD4+ T cell subsets between peritoneal fluid and peripheral blood in the three groups

The control group had increased numbers of Treg and Th2 cells in the PF compared to PB (both, P=0.046). This up-regulation was only detected in the follicular phase when compared with the luteal phase (Fig . 4).

In the early stages (I, II) of endometriosis, we observed increased numbers of Th2, Th17 and Treg cells in the PF compared to PB (P values were 0.005, 0.047 and 0.013, respectively). This observation was only noted in the follicular phase (P-values were 0.018, 0.028 and 0.018, respectively). Th17 cells were the only CD4+ T cell subsets that increased in the PF of stage III, IV endometriosis (P=0.013, Fig . 4). This change was also observed in the follicular phase (P=0.046). We did not observe any change in quantity of these T cell subsets between the PB and PF in the luteal phase in any of the groups.

## Discussion

Endometriosis is a complex disorder with variable immune disturbances (18, 19); however, the role of CD4+ T lymphocytes deviation in different stages of disease is not completely elucidated.

In this study, we compared the frequencies of Th1, Th2, Th17 and Treg cells between early and advanced stages of endometriosis, and in women without endometriosis, as a control group. Because the population of immune cells varies according to hormonal changes in the menstrual cycle (20), we also compared these cells in the follicular and luteal phases. We found no systemic deviation in frequency of CD4+ T subsets in different stages of endometriosis; however, Th17 cells were increased in the PF in patients with advanced endometriosis compared to controls.

First, we compared the numbers of four CD4+ T subsets in peripheral blood between the three groups. Our results did not show any significant differences in frequency of blood Th1, Th2, Th17 and Treg cells between the different groups. The systemic deviations of CD4+ T cell numbers between three groups were not reflected in our study. We found one study by Takamura et al. (18) that compared all four CD4+ T subsets concurrently in blood from patients with stage III, IV endometriosis (10 samples; 6 follicular and 4 luteal phases) with 10 normal individuals (4 follicular and 6 luteal phases). Similar to our results, they indicated no systemic changes in Th2, Th17 and Treg cells between the endometriosis and control subjects (18). However, these researchers confirmed an increased number of Th1 cells in the blood of patients with endometriosis (18). This finding differed from our results.

Regarding Th1 and Th2 cells, Szyllo et al. reported diminished IFN-γ levels and augmented IL-4 production by stimulated blood T cells of endometriosis patients; however, the authors did not divide the T cells into CD8 or CD4 cells (21). Also, the results of another study confirmed increased systemic CD4+IL-4+ and CD4+IL-10+ cells in patients with endometriosis compared to a control group (10). Our findings about circulatory Th2 frequencies were not consistent with these studies (10, 21).

In terms of blood Th17 cells, three independent studies demonstrated no significant differences in PB Th17 cell numbers between endometriosis and control groups (14, 18, 22), which supported our findings.

There are contradictory results concerning the frequency of Treg cells in blood samples between endometriosis and healthy women (11, 12, 18, 22). Although Olkowska- Truchanowicz et al. have reported decreased numbers of blood Treg cells in endometriosis (12), other investigators (11, 18, 22) reported that blood Treg cells remained unchanged between endometriosis and controls, which supported our results. Olkowska-Truchanowicz et al. compared the number of Treg cells in 17 blood samples obtained during the follicular phase from women with advanced stages (III, IV) of endometriosis to 15 samples from the follicular phase in control women without endometriotic foci. They introduced CD4+CD25highFoxP3+ cells as the Treg cell population (12). On the other hand, Gogacz et al. investigated the numbers of Tregs in two groups with endometriosis (15 samples in early stages and 7 samples with advanced endometriosis) and 20 women with unexplained infertility without any evidence of endometriosis. These researchers introduced CD4+CD25+FoxP3+ cells as Tregs and reported similar numbers of Treg cells between mild and advanced endometriosis (11). Khan et al. evaluated the frequency of Tregs amongst three groups – early stage endometriosis (n=15), advanced stage endometriosis (n=24) and control women (n=21), who were in different menstrual phases. Like other researchers, this group introduced CD4+CD25+FoxP3+ as Tregs and reported similar numbers of blood Tregs in endometriosis and control samples (22).

Next, we compared the number of different CD4+ T subsets locally in the PF. Th17 cells were the only CD4+ T cells increased in the PF of stage III, IV endometriosis relative to the control group. This trend was observed in both the follicular and luteal phases. We could not find any article that compared the number of all four CD4+ T subsets in the PF between endometriosis and control groups; however, Takamura et al. compared all four subsets in endometriotic and normal endometrial tissues, and reported increased Th17 cells in ectopic tissue compared to eutopic endometrium (18). Gogacz et al. compared the numbers of CD3+CD4+IL-17+ T cells between 22 infertile women with endometriosis (mild endometriosis n=15; severe endometriosis n=7) and 20 infertile women without any evidence of endometriosis. All samples were taken during the follicular phase. These researchers reported that the percentage of Th17 cells in the PF was higher in the higher stages of endometriosis (III, IV) compared to the lower stages (I, II) in the follicular phase; however, they did not observe any significant difference in the number of Th17 cells in the PF between the endometriosis and healthy groups. They concluded that a correlation existed between the frequency of Th17 in PF and severity of endometriosis (14). Chang et al. reported increased numbers of Th17 cells in the PF in endometriosis (stage I, II and stage III, IV) compared to control women. In comparison with other studies, the sample size of their study was more similar to our study and the results that pertained to the number of Th17 cells between stage III, IV and controls were concordant with our results (23). Our results suggested predominant Th17 responses in stage III, IV of endometriosis relative to the control group.

Th17 cells play prominent roles in induction of inflammation and development of endometriosis. IL17 stimulates secretion of IL-8 by endometrial stromal cells (ESCs), expression of cyclooxygenase 2 and proliferation of ectopic endometrial cells. IL-8 could induce the adhesion of stromal cells to fibronectin. In this way, IL-17 could increase endometriotic lesions (24-26).

Although some studies demonstrated increased levels of CD4+CD25+FoxP3+ cells in the PF (12, 22, 27) and ectopic tissue (28) of endometriosis subjects, we did not detect this increase in our study. On the other hand, some researchers reported unvarying numbers of Tregs in PF (11) or ectopic lesions (18, 29) in endometriosis, which supported our results.

We did not detect any significant difference in frequency of Th2 cells in the PF from different stages of endometriosis and controls; however, our results indicated higher numbers of Th2 cells in the PF exclusively in the luteal phase in the early stages of endometriosis compared to advanced stages. There are studies that illustrated deviation to Th2 immune responses, directly through investigating the expression of Th2 related cytokines or transcription factors (10, 30) or, indirectly (18) through deviation of Th1/Th2 proportions in endometriosis.

In the third step, we compared the frequency of different CD4+ T cells between the PF and PB in the different groups. We performed this comparison based on the menstrual cycle. We observed a fluctuation in CD4+ numbers between PB and PF only in the follicular phase. This finding could be related to the dominancy of oestrogen in the follicular phase and of progesterone in the luteal phase (31). Endometriosis is characterized by a dependency on oestrogen (32) and resistance to progesterone (33), which may guide us to observed alterations in follicular phase.

Our findings indicated that Th2 and Treg cells increased in the PF of the control group relative to blood. High doses of oestrogen could deviate immune responses from Th1 to Th2 (34). On the other hand, oestrogen levels increase from the pre-ovulatory to the end of the menstrual phase in the PF compared to blood (35, 36). Thus, in the last days of the follicular phase, which was compatible with the time of sampling, high peritoneal doses of oestrogen might cause shift of immune responses to Th2 (34). This could confirm the up-regulation of Th2 cells in the PF in control samples from the follicular phase. On the other hand, oestrogen could bind to its receptor on the surface of CD4+CD25-T cells and convert them to CD4+CD25+T cells that express FOXP3 and IL-10 (37). Increased oestrogen levels in PF in the pre-ovulatory phase could lead to upraised levels of Treg cells in PF compared to blood in the control samples. Compatible with our results, Khan et al. reported a modest increase in Treg cells in PF of normal subjects compared to PB (22).

The number of Th17 cells, as well as Th2 and Treg cells, in the early stages of endometriosis (stage I, II) were elevated in the PF compared to blood. The increased levels of Th17 cells in the PF compared to blood in stage (I, II) endometriosis could be explained by two assumptions. The first is related to production of C-C chemokine ligand 20 (CCL20) by ectopic endometrial cells. CCL20 is stimulated by inflammatory cytokines such as tumour necrosis factor-α (TNF-α) and IL-1β in the local microenvironment and it can attach to C-C chemokine receptor type 6 on Th17 cells, which leads to recruitment of these cells to the PF (19, 38). The second assumption is related to increased differentiation of naive T cells to Th17 at the local site of endometriotic tissue, which is rich in transforming growth factor beta (TGF-β), IL-23 and IL-6 (9, 39, 40).

With progression of endometriosis to stage III or IV, we found that only Th17 cells were increased in PF compared to PB. Similar to our findings, Gogacz et al. reported that there were elevated levels of Th17 cells in the PF of stage III, IV endometriosis, which was proportional to blood (14); however, other researchers reported no changes in the numbers of Th17 cells between the PF and PB in endometriosis subjects (22). It seems that Th17 cells could be involved in endometriosis development by production of inflammatory cytokines and angiogenic factors. Chang et al. confirmed that IL-27 from macrophages or ESCs could stimulate production of IL-10 by Th17 cells, which induces progression of endometriosis (23). In the higher stages of endometriosis, Th17 cells cause cyclooxygenase 2 induction, inflammation and cell adhesion. In this way, these cells could promote the development of endometriosis. We did not locate any article that compared all four subsets of CD4+ T cells in both PB and PF between early and advanced stages of endometriosis and controls. However, our study had some limitations such as the small sample size. In addition, we did not use anti-CD3 antibody for more specific determination of Th and Treg cells.

This study provided a comprehensive view of systemic and local changes in Th1, Th2, Th17 and Treg cells in early and advanced stages of endometriosis. Identification and application of local factors that affect Th17 cells can provide novel approaches for future treatments of advanced endometriosis.

## Conclusion

In this study, apparent changes in systemic CD4+ T cells were not found between different stages of endometriosis and the controls; however, our results indicated a predominance of Th17 cells in the local microenvironment of the PF. Th17 superiority could be related to disease progression because we observed a higher number of Th17 cells in the more advanced stages of endometriosis. However, future studies should be conducted to evaluate this relationship at the cellular and molecular levels.

## References

[1] Wang Y, Nicholes K, Shih IM (2020). The Origin and Pathogenesis of Endometriosis. Annu Rev Pathol.

[2] Parazzini F, Esposito G, Tozzi L, Noli S, Bianchi S (2017). Epidemiology of endometriosis and its comorbidities. Eur J Obstet Gynecol Reprod Biol.

[3] Greene AD, Lang SA, Kendziorski JA, Sroga-Rios JM, Herzog TJ, Burns KA (2016). Endometriosis: where are we and where are we going?. Reproduction.

[4] Vercellini P, Vigano P, Somigliana E, Fedele L (2014). Endometriosis: pathogenesis and treatment. Nat Rev Endocrinol.

[5] Lagana AS, Vitale SG, Salmeri FM, Triolo O, Ban Frangez H, Vrtacnik-Bokal E (2017). Unus pro omnibus, omnes pro uno: A novel, evidence-based, unifying theory for the pathogenesis of endometriosis. Med Hypotheses.

[6] Mier-Cabrera J, Jimenez-Zamudio L, Garcia-Latorre E, CruzOrozco O, Hernandez-Guerrero C (2010). Quantitative and qualitative peritoneal immune profiles, T-cell apoptosis and oxidative stress-associated characteristics in women with minimal and mild endometriosis. BJOG.

[7] Osuga Y, Koga K, Hirota Y, Hirata T, Yoshino O, Taketani Y (2011). Lymphocytes in endometriosis. Am J Reprod Immunol.

[8] Thiruchelvam U, Wingfield M, O'Farrelly C (2015). Natural killer cells: key players in endometriosis. Am J Reprod Immunol.

[9] Andreoli CG, Genro VK, Souza CA, Michelon T, Bilibio JP, Scheffel C (2011). T helper (Th)1, Th2, and Th17 interleukin pathways in infertile patients with minimal/mild endometriosis. Fertil Steril.

[10] Antsiferova YS, Sotnikova NY, Posiseeva LV, Shor AL (2005). Changes in the T-helper cytokine profile and in lymphocyte activation at the systemic and local levels in women with endometriosis. Fertil Steril.

[11] Gogacz M, Winkler I, Bojarska-Junak A, Tabarkiewicz J, Semczuk A, Rechberger T (2014). T regulatory lymphocytes in patients with endometriosis. Mol Med Rep.

[12] Olkowska-Truchanowicz J, Bocian K, Maksym RB, Bialoszewska A, Wlodarczyk D, Baranowski W (2013). CD4(+) CD25(+) FOXP3(+) regulatory T cells in peripheral blood and peritoneal fluid of patients with endometriosis. Hum Reprod.

[13] Podgaec S, Dias Junior JA, Chapron C, Oliveira RM, Baracat EC, Abrao MS (2010). Th1 and Th2 ummune responses related to pelvic endometriosis. Rev Assoc Med Bras.

[14] Gogacz M, Winkler I, Bojarska-Junak A, Tabarkiewicz J, Semczuk A, Rechberger T (2016). Increased percentage of Th17 cells in peritoneal fluid is associated with severity of endometriosis. J Reprod Immunol.

[15] de Barros IBL, Malvezzi H, Gueuvoghlanian-Silva BY, Piccinato CA, Rizzo LV, Podgaec S (2017). What do we know about regulatory T cells and endometriosis?. A systematic review. J Reprod Immunol.

[16] Berbic M, Fraser IS (2011). Regulatory T cells and other leukocytes in the pathogenesis of endometriosis. J Reprod Immunol.

[17] (1997). Revised American Society for Reproductive Medicine classification of endometriosis: 1996. Fertil Steril.

[18] Takamura M, Koga K, Izumi G, Hirata T, Harada M, Hirota Y (2015). Simultaneous Detection and Evaluation of Four Subsets of CD4+ T Lymphocyte in Lesions and Peripheral Blood in Endometriosis. Am J Reprod Immunol.

[19] Salmeri FM, Lagana AS, Sofo V, Triolo O, Sturlese E, Retto G (2015). Behavior of tumor necrosis factor-alpha and tumor necrosis factor receptor 1/tumor necrosis factor receptor 2 system in mononuclear cells recovered from peritoneal fluid of women with endometriosis at different stages. Reprod Sci.

[20] Lee S, Kim J, Jang B, Hur S, Jung U, Kil K (2010). Fluctuation of peripheral blood T, B, and NK cells during a menstrual cycle of normal healthy women. J Immunol.

[21] Szyllo K, Tchorzewski H, Banasik M, Glowacka E, Lewkowicz P, Kamer-Bartosinska A (2003). The involvement of T lymphocytes in the pathogenesis of endometriotic tissues overgrowth in women with endometriosis. Mediators Inflamm.

[22] Khan KN, Yamamoto K, Fujishita A, Muto H, Koshiba A, Kuroboshi H (2019). Differential levels of regulatory t cells and t-helper-17 cells in women with early and advanced endometriosis. J Clin Endocrinol Metab.

[23] Chang KK, Liu LB, Jin LP, Zhang B, Mei J, Li H (2017). IL-27 triggers IL-10 production in Th17 cells via a c-Maf/RORgammat/Blimp-1 signal to promote the progression of endometriosis. Cell Death Dis.

[24] Ahn SH, Edwards AK, Singh SS, Young SL, Lessey BA, Tayade C (2015). IL-17A contributes to the pathogenesis of endometriosis by triggering proinflammatory cytokines and angiogenic growth factors. J Immunol.

[25] Garcia-Velasco JA, Arici A (1999). Interleukin-8 stimulates the adhesion of endometrial stromal cells to fibronectin. Fertil Steril.

[26] Hirata T, Osuga Y, Hamasaki K, Yoshino O, Ito M, Hasegawa A (2008). Interleukin (IL)-17A stimulates IL-8 secretion, cyclooxygensase-2 expression, and cell proliferation of endometriotic stromal cells. Endocrinology.

[27] Podgaec S, Rizzo LV, Fernandes LF, Baracat EC, Abrao MS (2012). CD4(+) CD25(high) Foxp3(+) cells increased in the peritoneal fluid of patients with endometriosis. Am J Reprod Immunol.

[28] Berbic M, Hey-Cunningham AJ, Ng C, Tokushige N, Ganewatta S, Markham R (2010). The role of Foxp3+ regulatory T-cells in endometriosis: a potential controlling mechanism for a complex, chronic immunological condition. Hum Reprod.

[29] Scheerer C, Bauer P, Chiantera V, Sehouli J, Kaufmann A, Mechsner S (2016). Characterization of endometriosis-associated immune cell infiltrates (EMaICI). Arch Gynecol Obstet.

[30] Chen P, Zhang Z, Chen Q, Ren F, Li T, Zhang C (2012). Expression of Th1 and Th2 cytokine-associated transcription factors, T-bet and GATA-3, in the eutopic endometrium of women with endometriosis. Acta Histochem.

[31] Owen JA Jr (1975). Physiology of the menstrual cycle. Am J Clin Nutr.

[32] Kitawaki J, Kado N, Ishihara H, Koshiba H, Kitaoka Y, Honjo H (2002). Endometriosis: the pathophysiology as an estrogen-dependent disease. J Steroid Biochem Mol Biol.

[33] Patel BG, Rudnicki M, Yu J, Shu Y, Taylor RN (2017). Progesterone resistance in endometriosis: origins, consequences and interventions. Acta Obstet Gynecol Scand.

[34] Matalka KZ (2003). The effect of estradiol, but not progesterone, on the production of cytokines in stimulated whole blood, is concentrationdependent. Neuro Endocrinol Lett.

[35] Koninckx PR, Verhoeven G, Van Baelen H, Lissens WD, De Moor P, Brosens IA (1980). Biochemical characterization of peritoneal fluid in women during the menstrual cycle. J Clin Endocrinol Metab.

[36] Syrop CH, Halme J (1987). Peritoneal fluid environment and infertility. Fertil Steril.

[37] Tai P, Wang J, Jin H, Song X, Yan J, Kang Y (2008). Induction of regulatory T cells by physiological level estrogen. J Cell Physiol.

[38] Hirata T, Osuga Y, Takamura M, Kodama A, Hirota Y, Koga K (2010). Recruitment of CCR6-expressing Th17 cells by CCL 20 secreted from IL-1 beta-, TNF-alpha-, and IL-17A-stimulated endometriotic stromal cells. Endocrinology.

[39] Annunziato F, Cosmi L, Liotta F, Maggi E, Romagnani S (2008). The phenotype of human Th17 cells and their precursors, the cytokines that mediate their differentiation and the role of Th17 cells in inflammation. Int Immunol.

[40] Li S, Fu X, Wu T, Yang L, Hu C, Wu R (2017). Role of Interleukin-6 and Its Receptor in Endometriosis. Med Sci Monit.

